# Synthesis and antifungal activity of novel griseofulvin nanoparticles with zinc oxide against dermatophytic fungi: *Trichophyton mentagrophytes* and *Trichophyton verrucosum*: A primary study

**DOI:** 10.18502/cmm.8.2.10331

**Published:** 2022-06

**Authors:** Ali Abdul Hussein S. AL-Janabi, Abas Matrood Bashi

**Affiliations:** 1 Department of Microbiology, College of Medicine, University of Karbala, Iraq; 2 Department of Clinical Laboratories, College of AMS, University of Karbala, Iraq

**Keywords:** Dermatophytes, Griseofulvin, Nanoparticles, ZnO

## Abstract

**Background and Purpose:**

Dermatophytoses is an important type of skin disease caused by dermatophytes. The long-term treatment of this disease with standard antifungal agents may be improved through the application of nanotechnology. This study aimed to prepare nanoparticles of griseofulvin with zinc oxide and assess its antifungal action.

**Materials and Methods:**

Nanoparticles of griseofulvin with zinc oxide (GF-ZnO NPs) were prepared. Physical characteristics of new preparation and antidermatophytic action against two species of dermatophytes (*Trichophyton mentagrophytes* and *Trichophyton verrucosum*) were investigated. Testing of two species was considered a primary test for antifungals of griseofulvin nanoparticles.

**Results:**

Physical examination indicated that GF-ZnO NPs had typical nanoparticle characteristics. A new formulation showed effective inhibitory action against two fungal species with higher efficiency than that of griseofulvin. *T. mentagrophytes* required a higher MIC value (0.0625 µg/mL) of GF-ZnO NPs than that required by *T. verrucosum* (0.031 µg/mL).

**Conclusion:**

GF-ZnO NPs revealed an effective action against dermatophytes compared to griseofulvin alone. Nanoparticles containing griseofulvin may be used in the development of a novel drug for the treatment of dermatophytosis.

## Introduction

Dermatophytes are a significant group of fungi that produce a skin infection called dermatophytosis or tinea [ 1 ]. This group consists of three main genera: *Trichophyton* spp., *Microsporum* spp., and *Epidermophyton* spp. [ 2 ]. Griseofulvin is regarded as one of the most commonly used antifungals for the treatment of dermatophytosis [ 3 , 4 ]. Nanoparticles are successfully developed for this drug to improve its pharmaceutical potency [ 5 ]. The antidermatophytic effect of griseofulvin nanoparticles has been evaluated in some studies. Solid lipid nanoparticles loaded with griseofulvin in gel form have shown the good pharmaceutical capability for the treatment of guinea pigs infected with *Microsporum canis* [ 6 ]. Zinc-oxide nanoparticles (ZnO NPs) were found to have an antidermatophytic effect on several dermatophyte species [ 7 , 8 ]. Compared to standard antifungal agents, such as amphotericin B and miconazole, ZnO NPs also exhibited more inhibitory effects against dermatophytes [ 7 ]. Antidermatophytic effects of griseofulvin-ZnO nanoparticles (GF-ZnO NPs) were investigated against two species of dermatophytes.

## Materials and Methods

GF-ZnO NPs were prepared according to the methodology used by George et al. (2014) with some modifications in mixing two solutions [ 7 ]. The first solution consisted of 500mg of griseofulvin (Sigma-Aldrich, German) dissolved in 100mL of DMSO (5000 µg/mL). The second solution was prepared by dissolving 0.5g of Zn nitrate (Sigma-Aldrich, German) in 50 mL of distilled water (10000 µg/mL) in a conical flask and agitated for 3 h. A mixture of two solutions was placed in a shaker water bath at 70 o C for 18 h and then centrifuged and washed four times with deionized water. It was dried in an oven at 70oC to be crushed later. The liberation of griseofulvin from GF-ZnO NPs was tested at different pH levels using Lagergren equations (1898). 

The chemical bonds of the prepared GF-ZnO NPs were determined through Fourier transform infrared spectroscopy (FTIR) (Perkin-Elmer, 1725X Spectrophotometer, Japan) in a frequency range of 4000-400 cm^-1^. The specimen was pressed to a thin disc with KBr before measurement. The structure of the nanoparticles was investigated by X-Ray powder diffraction patterns (PXRD) (Shimadzu XRD-6000, X-ray diffractometer, Japan) at Cu-Kα QUOTE α spectrum using λ=1.540562 Å at 40 kW and 30 mA, with scan rate =1.0 degree/min. The diffractogram was recorded at 2θ 5-100°. Atomic Force Microscopy (AFM) images (Angstrom Advanced Inc., USA) were prepared to determine the shape of the nanoparticles. The topical analysis of surface roughness and the average square root roughness was measured using the equation adopted by Monef et al. (2013).

Fungi were isolated from a30 years old female infected with tinea corporis who attended the Al-Ammam Al-Hussein Medical City Hospital in Karbala province, Iraq, in July 2018. Scales from infected skin were cultured on Sabouraudʼs Dextrose agar (SDA, Himedia, India) with 0.05 g/L of chloramphenicol and incubated at 28ºC for 1-4 weeks. Fungi were primarily diagnosed as dermatophytes, according to morphological features of colonies and macroconidia [ 11 ]. A confirmatory diagnosis was made according to the molecular characteristics. The fungi DNA was extracted from the old cultivated mycelium (one week) by a spin column method using the EZ-10 spin column fungal genomic DNA mini-preps kit (Bio Basic INC., Canada). Polymerase chain reaction (PCR) was used for amplification of internal transcribed spacer regions (ITS) with primer pairs (ITS1andITS4) under reaction conditions mentioned earlier by White et al. (1990) [ 12 ]. The PCR for ITS was programmed at 94°C for 2 min, 35 cycles at 94°C for 35 sec, 52°C for 30 sec, and 72°C for 60 sec, with a final extension at 72°C for 6 min. Negative control was also applied in the absence of a DNA template. Fungal species were determined after sequencing by Bioneer (South Korea) and compared with other sequences stored in Gen Bank using the BLAST program.

The antifungal activity of GF-ZnO NPs was identified using the agar well diffusion method [ 13 ]. Various concentrations of GF-ZnO NPs (1, 0.5, 0.25, and 0.125 µg/mL) were prepared by dissolution in ethanol. A sterilized physiological saline solution containing 1.5 × 10^8^ cells/mL of fungus was prepared after adjustment to the 0.5 McFarland standard [ 14 ]. The fungi were inoculated on media by spreading 0.1 mL of fungal suspension on an SDA plate, and6 mm wells were then made by sterile cork borer. In total, 100 µL of GF-ZnO NPs solution was added to each well. Positive controls consisted of zinc nitrate dissolved in distilled water (71 mg/mL) and griseofulvin in ethanol (1 µg/mL), while distilled water and ethanol were used as negative controls. Cultures were incubated for 72 h at 37 °C and inhibition zones were measured. The MIC value for the new formulation was determined using the CLSI method, Should be modify [ 15 ]. A concentration of 2 × 10^4^ cfu/mL of fungi in Sabouraud Dextrose Broth (SDB) was prepared from a one-week-old colony. In plastic microtiter plates (96 wells), 100 µL of fungal inoculum was added to every single well. Two-fold concentrations of GF-ZnO NPs were prepared and 100 µL of each was added to inoculated wells. Two drug-free controls, including medium only and medium with fungi, were also used. Plates were incubated at 28°C and read visually after 3 days of incubation.

## Results and Discussion

**Figure 1:**
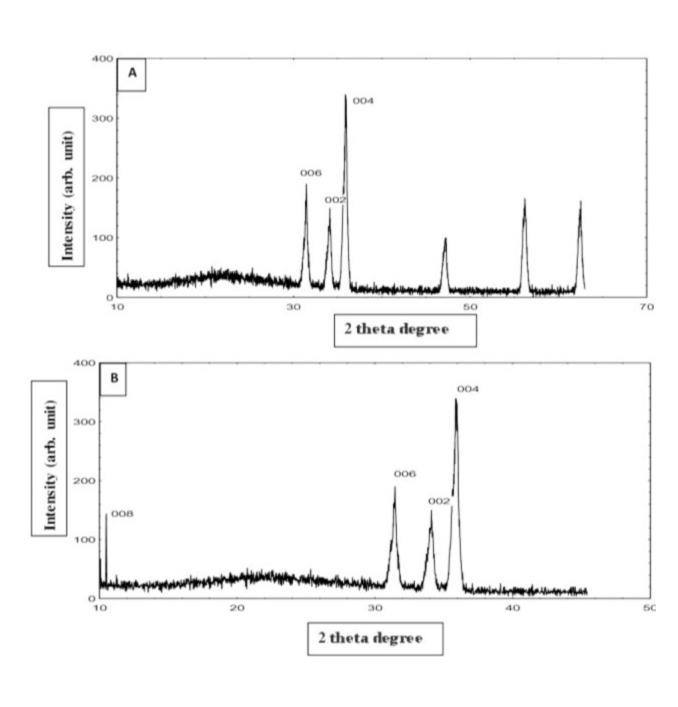
XRD image of: a- ZnO, b- GF-ZnO NPs

A review of the spectra of the FT-IR images revealed a significant change in the position of the griseofulvin bands after the ZnO intercalation. The intercalation of griseofulvin into ZnO, as presented in the XRD images, revealed a well-ordered phase of nanohybrid with a new peak at 2θ=10.4 for the new nanocompounds (Figure 1). The observation of other harmonics with the interlayer distance showed that griseofulvin found its path and assembled for the formation of monophasic nanohybrids, leading to the formation of well-defined nanohybrids. Meanwhile, the peaks belonging to ZnO were reduced in intensity compared to the more intensive model of griseofulvin interspersed with ZnO (Figure 1).

**Figure 2:**
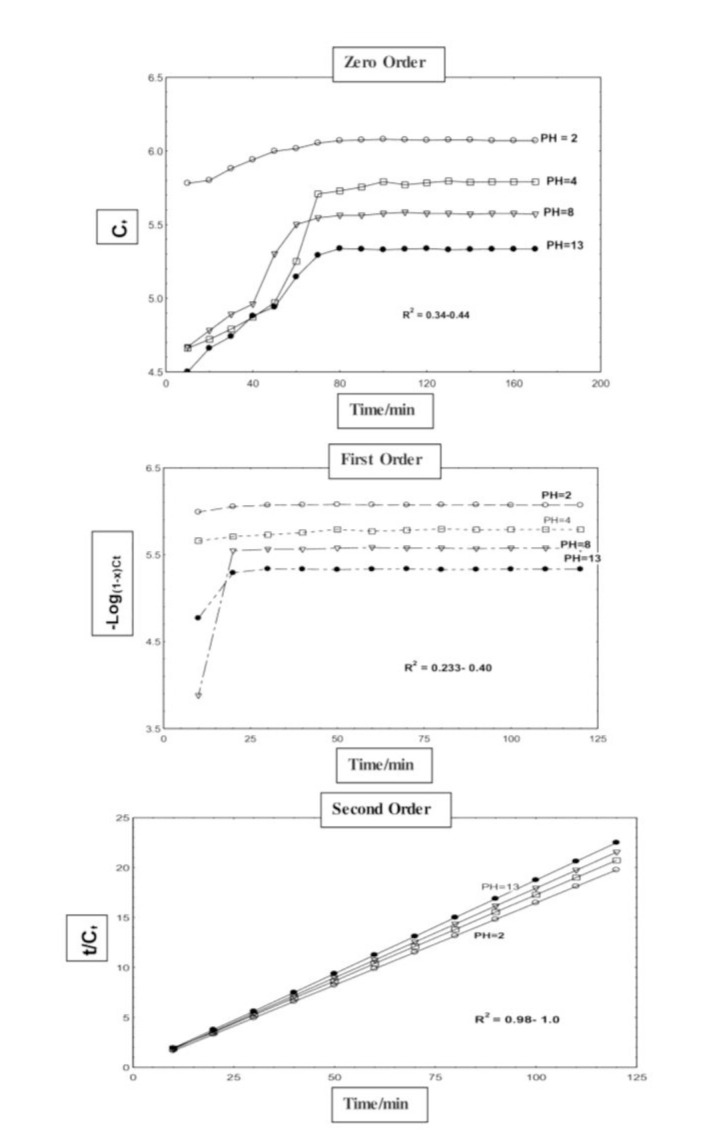
Zero-order, pseudo-first, and pseudo-second order model of the controlled-release

The AFM showed that the GF-ZnO NPs had a surface roughness of 13.68 nm, the mean square root was 11.68 nm, and the maximum surface was 195.56 nm. These values demonstrated the fully topographical appearance of the membrane surface. The release patterns of griseofulvin from the internal layers of GF-ZnO in aqueous solution at pH 2, 4.8, and 13 were 120,160, and 220 min, respectively. The second-order model was better fitted with r^2^ =1.0 (Figure 2). 

The antifungal activity of GF-ZnO NPs has been evaluated in two species of the genus *Trichophyton*. It was found that GF-ZnO NPs inhibit the growth of tested fungi more effectively than griseofulvin. The inhibition zone was increased with an increase in the concentration of GF-ZnO NPs. A significant effect was observed at a high concentration of GF-ZnO NPs (1 µg/mL) against *T. mentagrophytes* (21 mm) and *T. verrucosum* (23 mm). Zinc nitrate was found to have no effect on two dermatophytic fungi. *T. verrucosum* showed more sensitivity toward GF-ZnO NPs, compared to *T. mentagrophytes*. The MIC for GF-ZnO NP against *T. mentagrophytes* was also high (0.0625 µg/mL) compared to what is required for *T. verrucosum* (0.031 µg/mL)(P=0.04) (Table 1). 

**Table 1:** Inhibition zone and minimum inhibitory concentrations (MIC) of GF-ZnO NPs against dermatophytes

Compound	Concentration (µg/mL)	Inhibition zone (mm)	
		*T. verrucosum*	*T. mentagrophytes*
GF-ZnO NPs	1	21*, **	23*,**
	0.5	19.5*	21*
	0.25	15*	18*
	0.125	11*	14.5*
	MIC (µg/mL)	0.031	0.0625
Griseofulvin	1 µg/mL	15*	19*
	MIC (µg/mL)	0.09	0.28
Zinc nitrate	71 mg/mL	0	0

* Significant differences between zinc nitrate and other compounds at *P*<0.01

** Significant differences between fungi and griseofulvin at *P*<0.01

Nanotechnology is observed as an advanced field in the main dynamic examination areas of modern materials science [ 16 ]. Nanoparticles have new or improved properties that are enhanced by specific attributes such as size, circulation, and morphology. Based on analysis of the results of Fourier Transform Infrared (FT-IR) images, griseofulvin could form nanoparticles with ZnO. This was also clear with the results of powder X-ray diffraction that confirmed the presence of sharp diffraction peaks as an indicator of the well-ordered and highly crystalline structure of GF-ZnO NPs. Intercalation of an organic substance resulted in the expanded base spacing of ZnO caused by the size and spatial orientation of the organic substance in the interlamellar region of ZnO [ 17 ].

AFM is mainly used to analyze the surface topography and surface crystalline structure of formed nanoparticles. The results indicate that our new preparation has good crystalline uniformity and excellent homogeneity of GF-ZnO NPs [ 17 ]. The average rate of crystalline granularity is an indicator of membrane uniformity and potential use in solar cells [ 18 ]. The ion exchange kinetics is an indicator for the velocity rate of separation and diffusion of ions from the solid phase to the liquid phase. The reverse action may occur at the same time after overcoming all the interstitial and implied molecular forces that interfere with the ion exchange process in the solution. Ion exchange kinetics plays a very important role in determining the duration of the ion exchange process and the state of equilibrium in which the ion exchange process stops [ 19 ]. However, the ion exchange kinetics depends on several factors including; ion concentration, temperature, helper presence, rapid propagation of ions within the exchange, and the size of the ion exchange [ 20 ].

Griseofulvin is an effective antifungal used to treat different types of dermatophytosis, mainly for tinea capitis and onychomycosis [ 1 , 21 ]. Its mechanism of action in dermatophytic cells includes inhibition of cell division by blocking the formation of mitotic spindle fiber [ 22 ]. It usually takes a long time (usually 4-6 months) to treat these types of infections [ 21 ]. Another disadvantage of utilizing griseofulvin is that the tissue layers are poorly absorbed [ 21 , 22 ]. Therefore, the conversion of griseofulvin into nanoparticles could be considered the best solution for such problems. Miconization or nanocapsulation of griseofulvin was found to improve its digestion, absorption, bioavailability, and clinical efficacy [ 5 ]. On the other hand, ZnO NPs have also been shown to have antidermatophytic activity that can be considerably greater than standard antifungal agents [ 7 , 8 ]. Its antifungal action is primarily related to the generation of highly reactive ions or chemicals, such as OH-, H_2_O_2_, or O_2_, with the capacity to damage the fungal cell wall or plasma membrane [ 23 ]. Therefore, the combination of ZnO and griseofulvin can improve the antifungal ability of each, particularly when prepared at the nanoparticle level.

The standard effective MIC of griseofulvin for dermatophytes is 1 µg/mL [ 4 , 15 ]. Griseofulvin resistance has been reported in many species of dermatophytes, such as *T. verrucosum*, *T. mentagrophytes*, and *T. tonsurans* [ 3 ]. Therefore, mixing griseofulvin with other materials can increase its antifungal action without the need to increase concentration. In this study, it was demonstrated that GF-ZnO NPs had more significant effects with low MIC values for *T. mentagrophytes* and *T. verrucosum*. *T. mentagrophytes* showed a high MIC value of GF-Zn NPs compared to *T. verrucosum*. This variability in susceptibility is common in dermatophytes and may be related to differences in species [ 4 ].

Differences between fungal species may not be the only factor influencing the activity of antifungals. Concentration is also important in the evaluation of the antifungal activity. Increasing the concentration of the agent will increase its antifungal activity in most cases. This was demonstrated by the findings of the present study. The antifungal action of ZnO NPs against *T. mentagrophytes* and *Microsporum canis* was more effective at higher compared to lower concentrations [ 8 , 23 ]. However, the elevation of antifungal concentrations is not generally preferred since it reveals the inappropriate characteristics of the agent. Adverse effects of griseofulvin, such as headache and aplastic anemia, are usually increased when used at high concentrations [ 6 , 22 ]. Meanwhile, the toxicity rate of ZnO also rises at higher concentrations. Therefore, mixing these compounds in low concentrations will limit adverse traits and may show a synergistic effect against fungi.

## Conclusion

GF-ZnO NPs revealed a successful effect against dermatophytes, and its antidermatophytic effect works better in action, compared to a standard antifungal. Griseofulvin in the nanoparticle formulation could be used to develop a new medication used in the treatment of dermatophytosis.

## Acknowledgments

None.

## Authors’ contribution

The A.A.A. contributed in the study design, data collection, and writing the manuscript, and the A.M.B. contributed in the Methods section. 

## Conflicts of interest

The authors have no conflict of interest to declare.

## Financial disclosure

The authors received no funding for this research.

## Ethical Considerations

The study was approved by the Ethics committee of the College of Medicine, University of Karbala, Karbala, Iraq (No. 220), in March 2018.

## References

[1] Bouchara JP, Mignon B, Chaturvedi V ( 2017). Dermatophytes and dermatophytoses: A thematic overview of state of the art, and the directions for future research and developments. Mycopathologia.

[2] AL-Janabi AH ( 2014). Dermatophytosis: causes, clinical features, signs and treatment. J Symptoms and Signs.

[3] Gupta AK, Williams JV, Zaman M, Singh J ( 2009). In vitro pharmacodynamic characteristics of griseofulvin against dermatophyte isolates of Trichophyton tonsurans from tinea capitis patients. MedMycol J.

[4] da Silva Barros ME, de Assis Santos D, Hamdan JS ( 2007). Evaluation of susceptibility of Trichophyton mentagrophytes and Trichophyton rubrum clinical isolates to antifungal drugs using a modified CLSI microdilution method (M38-A). J Med Microbiol.

[5] Zili Z, Sfar S, Fessi H (2005). Preparation and characterization of poly-Ɛ-caprolactone nanoparticles containing griseofulvin. IntJ Pharm.

[6] Aggarwal N, Goundi S ( 2013). Preparation and in vivo evaluation of solid lipid nanoparticles of griseofulvin for dermal use. J Biomed Nanotechnol.

[7] George SA, Raj MS, Solomon D, Roselin P ( 2014). A comparative study of the anti-fungal activity of zinc oxide and titanium dioxide nano and bulk particles with anti-fungals against fungi isolated from infected skin and dandruff flakes. J Microbiol Biotechnol.

[8] EL-Diasty E, Ahmed MA, Okasha N, Mansour SF, EL-Dek SI, Abd EL-Khalek HM, Youssif MH ( 2013). Antifungal activity of zinc oxide nanoparticles against dermatophytic lesions of cattle. Rom J Biophys.

[9] Lagergran S ( 1898). Zur Theorie der Sogenannten Adsorption Geloster Stoffe Kungliga Svenska Vetenskapsakademiens. Handlingar.

[10] Monef RA, Asmiel RA, Mohmmed SG ( Pure Sci 2013). Study structural and optical properties of nanostructure ZnO thin film prepared by chemical bath deposited method. Tikrit J.

[11] Rippon JW (1988). Medical mycology.

[12] White TJ, Bruns T, Lee S, Taylor J, Innis MA, Gelfand DH, Sninsky JJ, White TJ (1990). Amplification and direct sequencing of fungal ribosomal RNA genes for phylogenetics. PCR protocols: A guide to methods and applications.

[13] Balouiri M, Sadiki M, Ibnsouda SK (2016). Methods for in vitro evaluating antimicrobial activity: A review. J Pharm Anal.

[14] Brown AE (2012). Bensons microbiological applications: laboratory manual in general microbiology.

[15] Indira G ( 2014). In vitro antifungal susceptibility testing of 5 antifungal agents against dermatophytic species by CLSI (M38-A) micro dilution method. Clin Microbial.

[16] Zak AK, Razali R, Majid WH, Darroudi M ( 2011). Synthesis and characterization of a narrow size distribution of zinc oxide nanoparticles. Int J Nanomedicine.

[17] Yessur NA (2011). Synthesis and characterization of nano hybrid compounds and kinetic controlled release study of 2, 4-dichloro and 4-chloro phenoxy acetate from zinc/aluminum layered double hydroxide by direct ion exchange method. [Masters of SciencesThesis].

[18] de Andrade JE, Rogério M, Andrade M ( 2016). AFM and XRD characterization of silver nanoparticles films deposited on the surface of DGEBA epoxy resin by ion sputtering. Polímeros.

[19] Ambrogi V, Fardella G, Grandolini G, Perioli L, Tiralti MC (2002). Intercalation compounds of hydrotalcite-like anionic clays with anti-inflammatory agents, II: Uptake of diclofenac for a controlled release formulation. AAPS PharmSciTech.

[20] Alemzadeh I, Vossoughi AM (2002). Controlled release of paraquat from poly vinyl alcohol hydrogel. Chem Eng Process.

[21] Reis Gavazzoni Dias MF, Pinto Quaresma-Santos MV, Bernardes-Filho F, da Fonseca Amorim AG, Schechtman RC, Azulay DR ( 2013). Update on therapy for superficial mycoses: review article part I. An Bras Dermatol.

[22] Suganthi M ( 2016). Antifungal agents and their action against dermatophytes: curious to know the facts. JInnov Pharm Biol Sci.

[23] Yehia RS, Ahmed OF ( 2013). In vitro study of the antifungal efficacy of zinc oxide nanoparticles against Fusarium oxysporum and Penicillium expansum. AfrJMicrobiol Res.

